# Neisseria leonii sp. nov., isolated from the nose, lung, and liver of rabbits

**DOI:** 10.1099/ijsem.0.006460

**Published:** 2024-07-18

**Authors:** Martin Boutroux, Sandrine Favre-Rochex, Olivier Gorgette, Gérald Touak, Estelle Mühle, Christiane Bouchier, Olivier Chesneau, Frédéric J. Veyrier, Dominique Clermont, Praveen Rahi

**Affiliations:** 1 Institut Pasteur, Université Paris Cité, Center of Biological Resources of Institut Pasteur (CRBIP), 75015 Paris, France; 2 Institut Pasteur, Université Paris Cité, Collection of Institut Pasteur (CIP), 75015 Paris, France; 3 Institut Pasteur, Université Paris Cité, Ultrastructural BioImaging Unit, 75015 Paris, France; 4 INRS-Centre Armand-Frappier Santé Biotechnologie, Bacterial Symbionts Evolution, Laval, Quebec H7V 1B7, Canada

**Keywords:** DNA uptake sequence, MALDI-TOF MS, *Neisseria*, phylogenomics, rabbit

## Abstract

A taxogenomic study of three strains (3986^T^, 51.81, and JF 2415) isolated from rabbits between 1972 and 2000 led to the description of a new *Neisseria* species. The highest sequence similarity of the 16S rRNA gene was found to *Neisseria animalis* NCTC 10212^T^ (96.7 %). The 16S rRNA gene similarity above 99 % and average nucleotide identity (ANI) values above 96 % among the strains, indicated that they belong to the same species. At the same time, the strains shared ANI values below 81 % and dDDH values below 24 % with all described *Neisseria* species. In the *bac120* gene phylogenetic tree, the three strains clustered near *Neisseria elongata* and *Neisseria bacilliformis* in the *Neisseria* clade. However, the *Neisseria* clade is not monophyletic, and includes the type strains of *Morococcus cerebrosus*, *Bergeriella denitrificans*, *Kingella potus*, *Uruburuella suis*, and *Uruburuella testudinis. Neisseria shayeganii* clustered outside the clade with members of the genus *Eikenella*. Amino acid identity (AAI) values were calculated, and a threshold of 71 % was used to circumscribe the genus *Neisseria*. According to this proposed AAI threshold, strains 3986^T^, 51.81, and JF 2415 were placed within the genus *Neisseria*. The cells of the three strains were Gram-stain-negative diplococcobacilli and non-motile. Optimal growth on trypticase soy agar occurred at 37 °C and pH 8.5 in aerobic conditions. Notably, all strains exhibited indole production in the API-NH test, which is atypical for *Neisseria* and the family *Neisseriaceae*. The strains exhibited a common set of 68 peaks in their MALDI-TOF MS profiles, facilitating the swift and accurate identification of this species. Based on genotypic and phenotypic data, it is proposed that strains 3986^T^, 51.81, and JF 2415 represent a novel species within the genus *Neisseria*, for which the name *Neisseria leonii* sp. nov. is proposed (type strain 3986^T^=R726^T^=CIP 109994^T^=LMG 32907^T^).

## Introduction

The family *Neisseriaceae* contains 20 genera with validly published names, as listed on the List of Prokaryotic names with Standing in Nomenclature (LPSN) website [1]. A recent genome sequence-based study refined the delineation of the family *Neisseriaceae* to 12 validly named genera at the time of the emended description of the family [2]. Additionally, two new genera, *Paralysiella* and *Wielerella*, have been recently described within this family [3,4]. Most *Neisseriaceae* members harbour unique genomic features known as DNA uptake sequences (DUS), which are specific to phylogenetic groups within this family [5]. Furthermore, the family *Neisseriaceae* exhibits diverse cell shapes, including cocci, bacilli, and also multicellular longitudinally diving prokaryotes within the genera *Conchiformibius*, *Alysiella* and *Simonsiella* [6]. The genus *Neisseria* serves as the type genus of the family *Neisseriaceae* and comprises 32 validly published species according to LPSN, including the important human pathogens *Neisseria gonorrhoeae* and *Neisseria meningitidis*. Notably, many *Neisseria* species have been isolated from a wide range of animals and a few are common in oral and nasopharyngeal microbiota in humans.

This study characterizes three *Neisseria* strains: 3986^T^, 51.81 and JF 2415. These strains were isolated from rabbits (unknown species) in Lyon (France) 1972, Tours (France) and Bern (Switzerland), respectively, and deposited at the Collection of Institut Pasteur (CIP) in 2002, 1987 and 2023. Culture collections provide quality-controlled microbial resources, often used as reference material or model systems for complex biological studies. These strains are expected to be valuable for future research on the ecology, evolution, and virulence of *Neisseria*. Additionally, as rabbits are common animal models, strains 3986^T^, 51.81 and JF 2415 will aid in studying host–microbe interactions. *Neisseria musculi* isolated from mice has been extensively used to study commensal colonization [7,8]. Furthermore, we performed preliminary work towards reorganizing the family *Neisseriaceae*, particularly the genera *Neisseria* and *Eikenella*, in addition to the description of a new species within the genus *Neisseria*.

## Methods

Strains 3986^T^ (=CIP 109994^T^) and 51.81 (=CIP 103045) were obtained from the CIP, while strain JF 2415 (=CCUG 45853=CIP 112445) was obtained from the Culture Collection University of Gothenburg (CCUG). Strain 3986^T^ (=R726^T^) was isolated at the Institut Pasteur Lyon (France) in 1972 from the liver of a baby rabbit, 51.81 was isolated by the Tours Departmental Veterinary Laboratory (France) in 1981 from the lung of a rabbit, and JF 2415 (=D320/00) was isolated in Bern (Switzerland) in 2000 from a rabbit nose. All strains were revived from the lyophilized vials on trypticase soy agar (TSA) at 30 °C for 24 h. Strains were stored for long term as lyophilized vials with 5 % *myo*-inositol and 10 % skimmed milk at 4 °C and with 15 % glycerol PBS at −80 °C. For comparative studies, *Neisseria bacilliformis* DSM 23338^T^ was obtained from DSMZ (German Collection of Microorganisms and Cell Cultures, Germany) and *Neisseria gonorrhoeae* CIP 79.18^T^, *Neisseria dentiae* CIP 106968^T^, *Neisseria elongata* CIP 72.27^T^ and *Kingella potus* CIP 108935^T^ from the CIP.

### 16S rRNA gene phylogeny

For 16S rRNA gene sequencing of strains 3986^T^ and 51.81, DNA was extracted using the InstaGenMatrix (Bio-Rad) and stored at −20 °C. The 16S rRNA gene was amplified using primers 27F and 1391R [9] and GoTaq DNA Polymerase (Promega). The PCR was performed as follows: initial denaturation at 95 °C for 3 min and then 30 cycles of denaturation (95 °C, 45 s), annealing (60 °C, 45 s), elongation (72 °C, 90 s). The amplified product was sent for Sanger sequencing at Eurofins Genomics (Ebersburg, Germany). The sequence quality was checked with BioEdit 7.2.5, and trimming and assembly were performed using CLCGenomicsWorkbench 20.0.4. For strain JF 2415, the 16S rRNA gene sequence deposited by CCUG was used (AY064548.1).

The NCBI nucleotide database was searched with blastn for 16S rRNA gene sequences of related strains. Additionally, rabbit microbiome data from a 2019 study [10] was also searched to find amplicon sequence variants (ASVs) identical to strain 3986^T^. Data was loaded on qiime2 2022.11 [11] with the q2-fondue 2022.2 plugin [12]. The appropriate trimming parameters were calculated with figaro 1.1.2 [13] and used with the Divisive Amplicon Denoising Algorithm 2 (dada2) [14] to produce the ASV table. blastn identities were calculated between these ASVs and the V4 region extracted from the 16S rRNA gene sequence of strain 3986^T^.

A reference dataset was built with the 16S rRNA gene sequences of all type strains of the 65 *Neisseriaceae* species listed on the LPSN (accessed on 12 February 2024) [1]. *Vitreoscilla filiformis* was excluded because it has been misclassified within the order *Neisseriales* [2]. All sequences were downloaded from the LPSN. Eight of them were replaced with sequences from EzBioCloud [15] because the LPSN sequences were less than 500 bp. The sequence of *Neisseria flava* was also replaced with its counterpart from EzBioCloud because the LPSN sequence shared 100 % identity with 16S rRNA gene sequence of *Moraxella caviae* CCUG 355^T^. See Table S1 for the detailed characteristics of the reference dataset. All 16S rRNA gene sequences were aligned using muscle [16] in mega 11 [17]. Phylogenetic trees were built using the maximum-likelihood (ML), neighbour-joining (NJ) and maximum-parsimony (MP) algorithms, with 1000 bootstrap replications. *Chromobacterium violaceum* ATCC 12472^T^ was used as an outgroup.

### Genome sequencing and phylogenetic analysis

For genome sequencing, DNA was extracted from the 24 h of growth on TSA for three strains using the Wizard Genomic DNA purification kit (Promega) and stored at −20 °C. The short-read sequencing was performed using a Nextseq 500 Instrument (Illumina) with a 2×150 bp paired-end protocol at the Plateforme de Microbiologie Mutualisée (P2M) of the Institut Pasteur. This was complemented with long-read sequencing using the Ligation sequencing gDNA kit (SQK-NBD114.24) and sequenced on a Mk1C device for 24 h (Oxford Nanopore Technologies) with an R10.4 flow cell produced using a Mk1C device (Oxford Nanopore Technologies). For the assemblies, all tools were used with default settings unless specified. Short reads were preprocessed with fastp 0.20.1 [18] and long reads with Filtlong 0.2.0 [19] (--min_length 1000 --keep_percent 95). Hybrid assemblies of strains 3986^T^ and JF 2415 were performed on Unicycler 0.4.8 with default settings [20]. The hybrid assembly of strain 51.81 was conducted with Trycycler version 0.5.0 [21] because Unicycler resulted three contigs instead of a complete genome. Genomic characteristics and quality were evaluated with CheckM 1.1.3 [22].

Average nucleotide identity (ANI) values were computed with FastANI 1.33 [23], and digital DNA–DNA hybridization (dDDH) values with formula 2 of the Type Strain Genome Server (TYGS) (https://tygs.dsmz.de/, accessed on 2 December 2023) [24]. A reference dataset was built using the whole genome sequences of the type strains of the *Neisseriaceae* species for which genome sequences were available. In total, 103 genomes were considered including those of strains 3986^T^, 51.81 and JF 2415, as well as 34 genomes listed in the GTDB (Genome Taxonomy Database) as new genera and/or species of *Neisseriaceae* but not formally described [25]. The genome of *Chromobacterium violaceum* ATCC 12472^T^ was used as an outgroup (see Table S2 for the full list of genomes). We used GTDBTk 2.1.1 [26] to identify, extract and align concatenated predefined and phylogenetically informative markers (*bac120* markers) to perform a taxonomy study (see Table S3 for the list of genes comprised in *bac120*). Ribosomal genes were identified, extracted and aligned using the rMLST (ribosomal multilocus sequence typing) scheme from BIGSdb [27]. Both alignments were used to build ML phylogenetic trees using iq-tree 2.2.2.2 [28]. The reconstruction of phylogenetic trees involved the selection of the best method by ModelFinder [29] and 1000 ultrafast bootstrap replications. The EzAAI 1.2.3 pipeline [30] was used to calculate AAI values clustering proteins using clustering thresholds of 40 % amino acid identity and 50 % coverage length. Results were plotted with the ggplots module in R.

Annotation step was performed with the Prokaryotic Genome Annotation Pipeline (PGAP 2023-05-17) [31]. Genomes were submitted to VFanalyzer (www.mgc.ac.cn/VFs/, accessed on 26 February 2024) [32] to identify virulence factors and resistance genes were identified using the the Resistance Gene Identifier (RGI) tool [33] on CARD (https://card.mcmaster.ca/analyze/rgi, accessed on 26 February 2024). The biosynthetic gene clusters for secondary metabolites were found by using antiSMASH (https://antismash.secondarymetabolites.org/https://antismash.secondarymetabolites.org/#!/start, accessed on 26 February 2024) [34]. The count function of the Jellyfish 2.3 module [35] was used to count the number of repeats of size 12 kmers. Type-four pili systems were identified with MacSyFinder 2.1.3 using the TFFscan models [36]. Furthermore, anti-phage defense systems were detected in the genome of strains by submitting the genomes to DefenseFinder’s web page [37].

### MALDI-TOF MS-based identification

Whole-cell proteins were extracted using ethanol–formic acid from the bacterial cultures after 24 h of growth on TSA, to generate the mean spectral profile (MSP). Protein profiles were generated for each strain ranging from 2 to 20 kDa by matrix-assisted laser desorption/ionization time-of-flight mass spectrometer (MALDI-TOF MS) Biotyper sirius (Bruker Daltonik GmbH). A total of 27 replicate spectra were generated for each strain and used to create the MSP [38,39]. After preliminary screening, protein spectra were processed with Clover MS Data Analysis software (https://platform.clovermsdataanalysis.com/login, accessed on 12 February 2024). To identify common peaks, the spectra were aligned using a linear mass tolerance of 600 ppm. Peaks present in all strains were selected as biomarker peaks, and the peak list was exported.

### Phenotypic features

The subcommittee of the International Committee on Systematics of Prokaryotes (ICSP) for the family *Neisseriaceae* was dissolved due to its inactivity [40]. Therefore, we performed the phenotypic characterization based on recent *Neisseria* species descriptions [41,44]. All phenotypic tests were done in-house for strains 3986^T^ and 51.81, while results concerning strain JF 2415 were obtained from CCUG (https://ccug.se/strain?id=45853, accessed on 11 September 2023) unless specified otherwise.

Strains 3986^T^ and 51.81 were grown on different growth media, including TSA, Columbia agar with 10 % horse blood, brain heart infusion (BHI), and chocolate agar supplemented with PolyVitex (bioMérieux). The temperature range for growth was determined using TSA plates incubated at different temperatures (4, 15, 28, 37 and 45 °C). All later incubations were done at 37 °C unless mentioned otherwise. Tryptone soya broth (TSB) with different pH (4, 5.5, 7, 8.5, 10, 11 and 12) set by adding HCl and NaOH before autoclaving was used to estimate the growth in different pH. Similarly, TSB pH 7 with different concentrations of Oxgall (0.1, 0.3 and 0.5 %) was used to determine the bile salt tolerance. Growth was measured by measuring absorbance at 600 nm with the Infinite M Nano+ (tecan). Incubation under microaerophilic and anaerobic conditions were tested using round 2.5 l jars with CampyGen and AnaeroGen products (ThermoFisher).

Microscopic observations were performed to determine the cell shape, size and motility of cultures grown on BHI medium. Motility was determined examining a small drop of bacterial culture in the centre of a microscope slide with the wet mount method [45]. Gram-staining was performed by using Aerospray Gram (ELITechGroup). For electronic microscopy, the bacterial cells were fixed to 300-mesh Formvar-Cu-coated grids (Electron Microscopy Sciences) for 15 min. The grids with cells were placed on a drop of ultrapure water and transferred to a drop of 2 % glutaraldehyde in 0.1 M sodium cacodylate buffer for 10 min at room temperature. After rinsing with ultrapure water, grids were stained for 15 s with 2 % aqueous uranyl acetate and dried. Images were captured with Tecnai Spirit 120Kv transmission electron microscope (TEM) equipped with a bottom-mounted Eagle 4kx4k camera (FEI).

Cultures for biochemical tests were grown using TSA plates incubated at 37 °C for 24 h unless specified otherwise. Oxidase activity was determined by putting a smear of culture on an oxidase strip (Sigma-Aldrich), and catalase activity was measured recording the bubble formation by bacterial cells, in response to hydrogen peroxide solution (Sigma-Aldrich).

The strains for comparative tests were chosen based on their closeness to strains 3986^T^ and 51.81 in ANI and TYGS (*N. dentiae* CIP 106968^T^), as well as in the *bac120* tree (*K. potus* CIP 108935^T^, *N. bacilliformis* DSM 23338^T^ and *N. elongata* CIP 72.27^T^). Type species *N. gonorrhoeae* CIP 79.18^T^ was also included. Physiological characterization of the strains was performed with the API NH and API ZYM test systems according to the instructions of the manufacturer (bioMérieux). For the API NH tests, all strains were grown on chocolate agar, while for the API ZYM test only *N. gonorrhoeae* CIP 79.18^T^ was grown on chocolate agar as the rest could grow on TSA. Reduction of nitrate and nitrite was evaluated by adding the reagents NIT 1 and NIT 2 (bioMérieux) as well as zinc in the overnight-grown cultures in nitrate and nitrite broth. Antibiotic susceptibility was determined using the disc diffusion method on Mueller–Hinton agar for 16 antibiotics following the recommendations of Comité de l’antibiogramme de la Société Française de Microbiologie (CA-SFM)/European Committee on Antimicrobial Susceptibility Testing (EUCAST) [46].

## Results and discussions

### 16S rRNA analysis and phylogeny

The 16S rRNA genes of strains 3986^T^ and 51.81 sequenced by Sanger method were 1254 bp and 1263 bp long, respectively, while 1536 bp long sequences were extracted from the genome sequences of both strains. The sequences originated from different sequencing methods shared 100 % identity. Therefore, we used the 16S rRNA gene sequences extracted from genomes for all database searches and phylogenetic analysis. The 16S rRNA gene sequence of strain 3986^T^ shared 99.09 and 99.05 % identity with strains 51.81 and JF 2415, respectively, above the threshold for species delineation of 98.65 % [47].

As all three strains were isolated from rabbits, we investigated their proximity to rabbits by searching for identical ASVs in available 16S rRNA gene amplicon data from rabbits [12,48]. We could not use the sequence data from one study due to an invalid accession number (PRJCA006344) and a lack of author response [48]. However, we used gut microbiome data from wild European rabbits (SRP129755) [12]. We found one ASV that was 100 % identical to strain 3986^T^, and was present 19 times in one of the 126 samples (SRR6475038) (metadata available in the Supplementary material). The strains were isolated from the nose, liver, and lung of rabbits, and their lower occurrence in the faecal microbiome suggests specificity to certain organs. It should be noted that the 16S rRNA gene V4 region (253 nucleotides) of *Kingella potus* type strain and strain 3986^T^ were 100 % identical, so this analysis applies to both *K. potus* strain 3/SID/1128^T^ and strain 3986^T^.

**Fig. 1.**
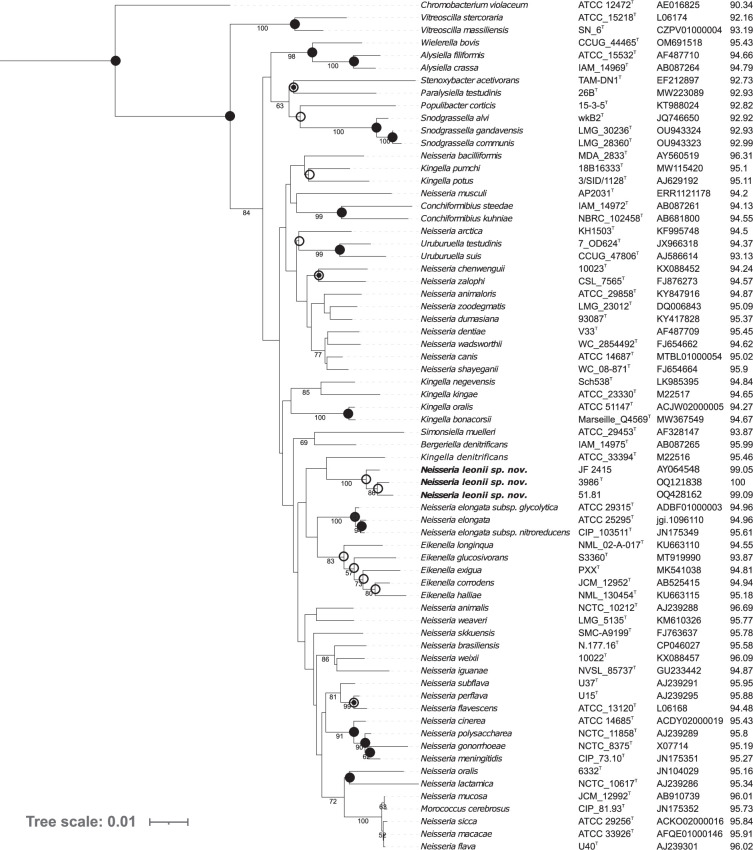
16S rRNA gene phylogenetic tree obtained in mega 11 with the neighbour-joining method from the sequences of strains 3986^T^, 51.81, JF 2415 and all type strains of described *Neisseriaceae* species. 1256 sites were used to build the phylogenetic tree with using “complete deletion” option. Last column shows % of identity with the 16S rRNA gene sequence of strain 3986^T^. Empty circles indicate branches that were also found using the maximum-likelihood method, circles with a dot indicate branches that were also found using the maximum-parsimony method and filled circles indicate branches that were also found using both methods. The Tajima–Nei method with complete deletion was used for data treatment. *Chromobacterium violaceum* ATCC 12472^T^ was used as an outgroup. Bootstrap values above 50% are displayed. Bar, 0.01 changes per site.

A blastn search between the 16S rRNA gene sequence of strain 3986^T^ and the reference dataset (Table S1) resulted in 46 *Neisseriaceae* species with 16S rRNA gene sequence similarity threshold higher than 94.5 % [48]. These species belong to the genera *Neisseria*, *Kingella*, *Alysiella*, *Bergeriella*, *Conchiformibius*, *Eikenella*, *Morococcus*, and *Wielerella*, making it difficult to assign strain 3986^T^ to a specific genus based solely on 16S rRNA gene similarity. The highest similarity was to *Neisseria animalis* NCTC 10212^T^ (96.7 %). In all phylogenetic trees based on 16S rRNA gene sequences, strains 3986^T^, 51.81 and JF 2515 formed a distinct clade. This clade clustered with *Kingella denitrificans* in the NJ tree, but this position was not consistent in the ML and MP trees (Fig. 1). It should be noted *Neisseria lisongii* and *N. yangbaofengii* were not included in this analysis as they were described later, and their 16S rRNA gene identity with strain 3986^T^ was relatively low (95.62 and 95.93 %, respectively). We observed that members of *Neisseria* were distributed across several clades, with members of genera *Bergeriella*, *Conchiformibius*, *Eikenella*, *Kingella*, *Morococcus*, *Simonsiella*, and *Uruburuella* embedded within these non-monophyletic clades. The members of *Kingella* were spread over three clusters, complicating the taxonomic classification of strains 3986^T^, 51.81 and JF 2515.

### Genome features

Complete genomes were obtained for all three strains after hybrid assembly. The genome sequence of strains 3986^T^, 51.81 and JF 2415 were 2.21, 2.32 and 2.23 Mbp long, respectively, consistent with typical sizes of *Neisseria* type strains (median, 2.43 Mbp; range, 1.83 and 2.93 Mbp). The chromosome of strain 51.81 was notably longer due to the presence of a 79 kbp prophage. The DNA G+C contents were 56.88, 56.92 and 56.98 mol%, respectively, falling within the upper range of G+C content observed in *Neisseria* species (median, 51.0 %; range, 44.6–59.4 %). Strain 3986^T^ exhibited ANI values of 96.91 and 97.08 %, and dDDH values of 70.7 and 71.8 % when compared to strains 51.81 and JF 2415, respectively. These values surpass the species delineation thresholds of 95–96 % for ANI and 70 % for dDDH [48]. *Neisseria dentiae* DSM 19151^T^ was the closest species for strain 3986^T^ with ANI and dDDH values, 79.89 and 23.8 %, respectively (see Table S4 for detailed results).

**Fig. 2.**
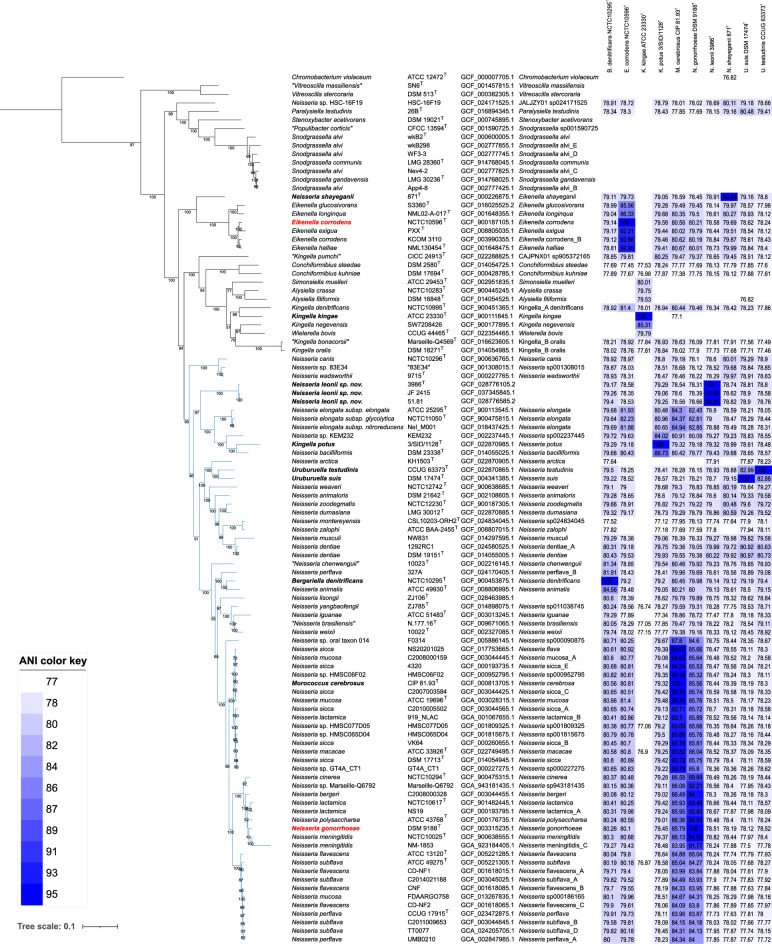
Phylogenetic tree obtained with iq-tree from the alignment of the *bac120* genes of strains 3986^T^, 51.81, JF 2415 and 100 type strains of the *Neisseriaceae* family with the maximum-likelihood method. Each line contains the isolate name, the accession number, the name of the species according to LPSN and according to GTDB if it differs, and a heatmap depicting the ANI values with *B. denitrificans* NCTC 10295^T^, *E. corrodens* NCTC 10596^T^, *K. kingae* ATCC 23330^T^, *K. potus* NCTC 13336^T^, *M. cerebrosus* CIP 81.93^T^, *N. gonorrhoeae* DSM 9188^T^, *N. leonii* 3986^T^, *N. shayeganii* 871^T^, *U. suis* DSM 17474^T^ and *U. testudinis* CCUG 63373^T^. The taxonomy of these strains and of strains 51.81 and JF 2415 is in bold to highlight them. Type species of *Neisseria* and *Eikenella* are also coloured in red. The *Neisseria* cluster is highlighted in blue. The Q.insect+F+I+R6 model was chosen according to the Bayesian information criterion information by ModelFinder. *Chromobacterium violaceum* ATCC 12472^T^ was used as an outgroup. Bootstrap values above 50% are displayed. Bar, 0.1 changes per site.

We found that the core-gene tree based on the *bac120* gene set is more discriminatory than the rMLST tree (Figs 2 and S1, available in the online Supplementary Material). Even though the core-gene (*bac120*) tree consists of more monophyletic clusters than the 16S rRNA gene trees and the rMLST tree, it still had a few non-monophyletic clusters. Particularly, the members of *Neisseria* were grouped with *Morococcus cerebrosus*, *Bergeriella denitrificans*, *Kingella potus, Uruburuella suis* and *U. testudinis* while *Neisseria shayeganii* was placed close to the *Eikenella* clade. The placement of three strains along with all subspecies of *Neisseria elongata* outside the main *Neisseria* cluster in rMLST phylogeny was noticed, while the *bac120* gene phylogeny placed all *Neisseria* species except *N. shayeganii* including the strains 3986^T^, 51.81 and JF 2415. Although rMLST was previously considered the best method for classifying *Neisseria* [49], this result is expected since the *bac120* gene set extends the rMLST set by including non-ribosomal genes. Similar clustering of members of different genera was identified previously in ML trees based on the alignment of 596 core proteins [2,6, 50].

**Fig. 3.**
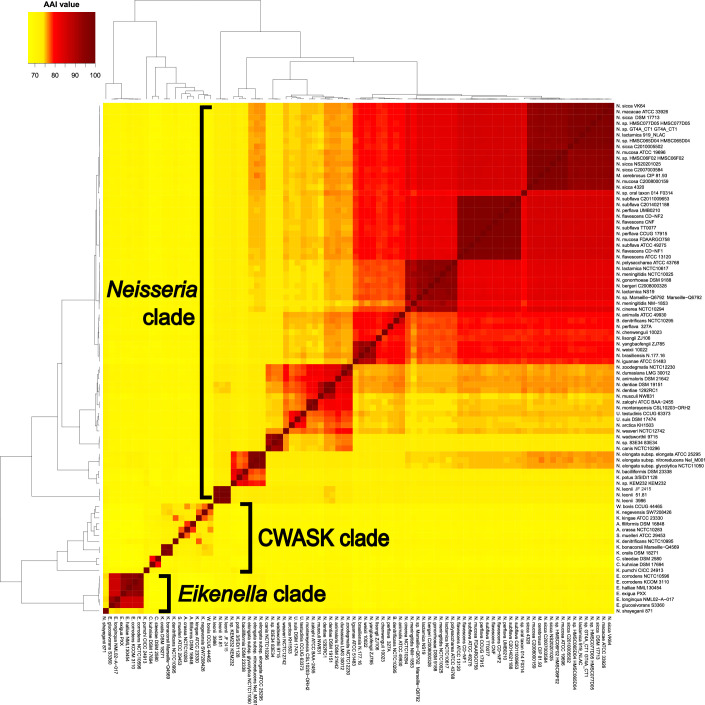
Pairwise AAI values between whole genome sequences of *Neisseria leonii* strains 3986^T^, 51.81, JF 2415 and type strains of species from *Alysiella*, *Bergeriella*, *Conchiformibius*, *Eikenella*, *Kingella*, *Morococcus*, *Neisseria*, *Simonsiella*, *Uruburuella*, and *Wielerella*. The clustering between rows and columns was done with 1-Pearson correlation coefficient and the UPGMA agglomeration method.

**Fig. 4.**
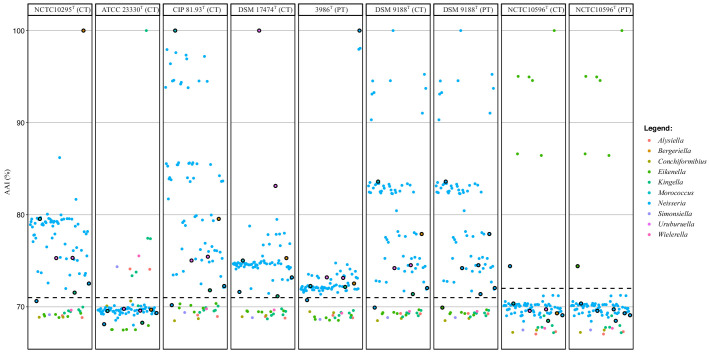
Average amino acid identity (AAI) values between the genomes of the dataset and the type species of the studied genera, namely *B. denitrificans* NCTC 10295^T^, *K. kingae* ATCC 23330^T^, *M. cerebrosus* CIP 81.93^T^, *U. suis* DSM 17474^T^, *N. gonorrhoeae* DSM 9188^T^, *E. corrodens* NCTC 10596^T^ plus *N. leonii* sp. nov. 3986^T^. Black circles and letters highlight the new species and the propositions of reclassification/reunification: *N. shayeganii* (A), *M. cerebrosus* (B), *U. suis* DSM 17474^T^ (C), *U. testudinis* (D), *K. potus* (E), B. denitrificans (F), *N. leonii* (G). CT stand for current taxonomy and PT for proposed taxonomy. Dashed lines indicate proposed values of AAI thresholds to delineate the genera: 71 for *Neisseria* and 72 for *Eikenella*.

AAI values were computed between 88 genomes, encompassing all species of the family *Neisseriaceae* except *Paralysiella*, ‘*Populibacter’*, *Snodgrassella*, *Stenoxybacter* and *Vitreoscilla*, which were distantly related to the other genera in the core-gene tree. The resolution of AAI values to delineate prokaryotes above the species level has been considered better than ANI values [30]. We observed three main clusters in the AAI matrix (Fig. 3) corresponding to *Neisseria*, *Eikenella* and the CWASK clade. The latter was left aside as its taxonomy was thoroughly investigated in a recent study [51]. As anticipated, *N. shayeganii* 871^T^ clustered with type species *E. corrodens* NCTC 10586^T^ and other members of *Eikenella* species. Conversely, strains 3986^T^, 51.81 and JF 2415, along with *U. suis* DSM 17474^T^, *U. testudinis* CCUG 63373^T^, *K. potus* NCTC 13336^T^, *B. denitrificans* NCTC 10295^T^ and *M. cerebrosus* CIP 81.93^T^ clustered with the type species *N. gonorrhoeae* DSM 9188^T^ and other members of *Neisseria* species. Considering that *N. shayeganii* belongs to *Eikenella* and the other five problematic species belong to *Neisseria*, AAI thresholds of 72 and 71 % accurately delineated the genera *Eikenella* and *Neisseria*, respectively (Fig. 4).

Most *Neisseriaceae* species exhibit natural competence, a shared ability for transformation facilitated by the presence of a small 12-mer known as DUS on extraneous DNA in the environment [5]. These DUS sequences exhibit specific dialects unique to different species or genera, with eight distinct dialects identified thus far. These dialects, which are highly prevalent among *Neisseriaceae* genomes, often correlate with the phylogeny of *Neisseriaceae* species based on core genome sequences [5]. This specificity can be harnessed to identify mobile genetic elements within genomes, as variations in DUS sequences may indicate the presence of sequences specific to other species [52].

Frye *et al*. [5] identified four predominant DUS dialects – AG-DUS, AT-DUS, AG-mucDUS and TG-wadDUS – among *Neisseria* species. In contrast, AG-eikDUS is characteristic of *Eikenella* species. A comprehensive analysis of all 12-mer sequences repeated more than 100 times across the genomes in our dataset aligned with these previous findings [5]. Notably, AG-DUS was the dominant dialect in strains 3986^T^, 51.81 and JF 2415, and for *Bergeriella denitrificans*, *Kingella potus*, and both *Uruburuella* species (ranging from 3935 to 1624 repetitions), while AG-mucDUS was the dominant dialect in *Morococcus cerebrosus* (2463 repetitions) (Table S5). Furthermore, AG-eikDUS dominated in *Neisseria shayeganii* (1661 repetitions), like other *Eikenella* species. Additionally, seven 12-mer sequences, characterized by the sequence CTG in positions 6–8, were found more than 100 times in at least one genome, suggesting potential new dialects.

**Fig. 5.**
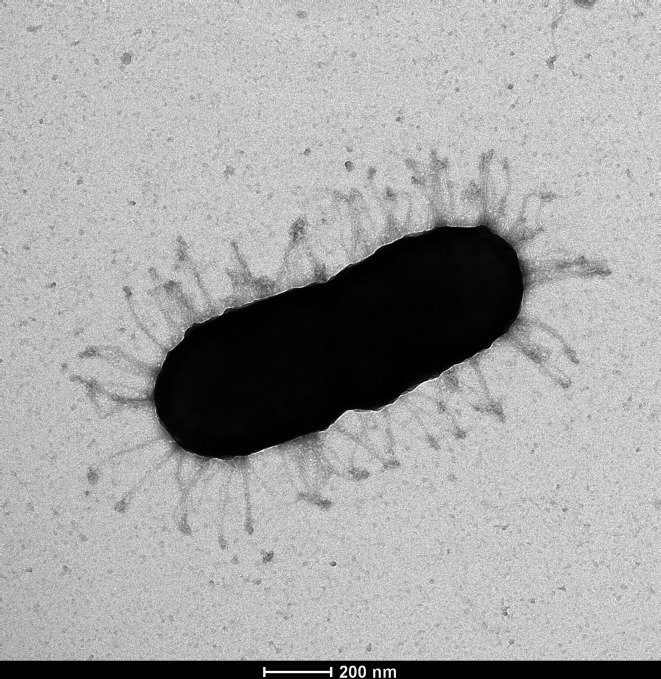
Transmission electron microscopy image of *Neisseria leonii* sp. nov. 3986^T^. The cells are surrounded by Tfp-like fibres. Scale bar 200 nm.

The competence for DNA transformation of *Neisseriaceae* species is possible via a Type IV Pili (Tfp) like in most Gram-negative bacteria. The *comP* pseudopilin on the bacterial surface can recognize environmental DNA fragments harbouring a DUS [53]. Once the recognized DNA element binds to *comP*, it enters the bacterium via the retraction of *comP* through the action of the *pilT* motor protein. Tfp-like fibres were indeed visible on the TEM images of strain 3986^T^ (Figs 5 and S2). MacSyFinder identified a complete type-four pili system in all three strains (six mandatory genes *pilA*, *pilB*, *pilC*, *pilD pilT*, *pilQ* plus five accessory genes). The gene *comP* was not identified by MacSyFinder but a blastp comparison between the sequence A0A125WA94 of Uniprot associated with *Neisseria meningitidis* and the CDS identified by PGAP gave a hit for the three strains with 96 % coverage for 36.73 % identity. Our data indirectly supports the hypothesis that strains 3986^T^, 51.81 and JF 2415 may be transformable as can be expected from a member of the family *Neisseriaceae*.

Although the isolation source for all three strains was rabbits, there is no available information regarding the health status of these rabbits. The genomic analysis revealed the presence of 34 virulence factors in strain 3986^T^, 35 in strain 51.81, and 36 in strain JF 2415, with 33 factors being common among them (Table S6). This substantial number of virulence factors suggests the genetic potential of these strains as potential pathogens. It is noteworthy that a majority of these virulence factors, specifically 22 in strain 3986^T^ and 23 in strains 51.81 and JF 2415, were identified in the *Neisseria* database of VFanalyzer. The remaining factors were reported in various bacterial genera. These virulence factors play crucial roles in adhesion (four associated with the type IV pili system), iron uptake (six to seven), and stress adaptation (five). Additionally, commensal *Neisseria* species have been known to act as reservoirs for resistance genes and virulence factors that can be acquired by pathogenic *Neisseria* species [54,55].

Strain 3986^T^ also contained a salmochelin while strain 51.81 contained a ferric enterobactin transport protein A/ferric-repressed protein B and a nitrate reductase. Strain JF 2415 contained both genes. This coincided with the phenotypic characteristics performed which proved only strains 51.81 and JF 2415 could perform denitrification (Table 1). Reduction of nitrate and nitrite is a typical feature for most *Neisseria*, enabling the bacteria to thrive in environments with low oxygen levels [56]. Additionally, four putative secondary metabolite clusters were identified in the genomes of the three strains using anti-SMASH pipeline. These include hserlactone, facilitating communication between fungi and bacteria [57], resorcinol, employed in various products from rubber to antiseptics, arylpyolene, a pigment providing protection against reactive oxygen species [58], and terpene, a diverse class of natural products with various roles in mediating antagonistic and beneficial interactions among organisms [59]. Furthermore, diverse anti-phage defense systems were identified among the genomes of the three strains (Table S7). Eleven anti-phage systems were identified for strain 51.81, nine for strain JF 2415 and five for strain 3986^T^. Notably, multiple copies of restriction-modification (RM) Type II were identified for all strains, consistent with RM systems being the most prevalent antiphage defense systems [60,61].

**Table 1.** Biochemical features of strains 3986^T^, 51.81 and JF 2415 in comparison with the phylogenetically closely related species from the genera *Neisseria* and *Kingella* Strains: 1, 3986^T^; 2, 51.81; 3, JF 2415; 4, *N. bacilliformis* DSM 23338^T^; 5, *K. potus* CIP 108935^T^; 6, *N. dentiae* CIP 106968^T^; 7, *N. elongata* CIP 72.27^T^; 8, *N. gonorrhoeae* CIP 79.18^T^. All data from this study. nd, No data.

Characteristic	1	2	3	4	5	6	7	8
Source of isolation	Rabbit, liver	Rabbit, lung	Rabbit, nose	Human, submandibular wound	Human, wound from Kinkajou bite	Cow, dental plaque	Human, pharynx	Unknown
Glucose	−	−	−	+	−	+	−	+
Fructose	−	−	−	−	−	+	−	−
Saccharose	−	−	−	−	−	+	−	−
Lipase	+	−	−	−	−	−	−	−
Proline arylamidase	−	+	+	+	−	−	+	+
Indole	+	+	+	−	−	−	−	−
Nitrate reduction	−	+	+	+	+	+	−	nd
Nitrite reduction	−	+	+	+	+	+	+	nd

### MALDI-TOF MS-based typing

**Fig. 6.**
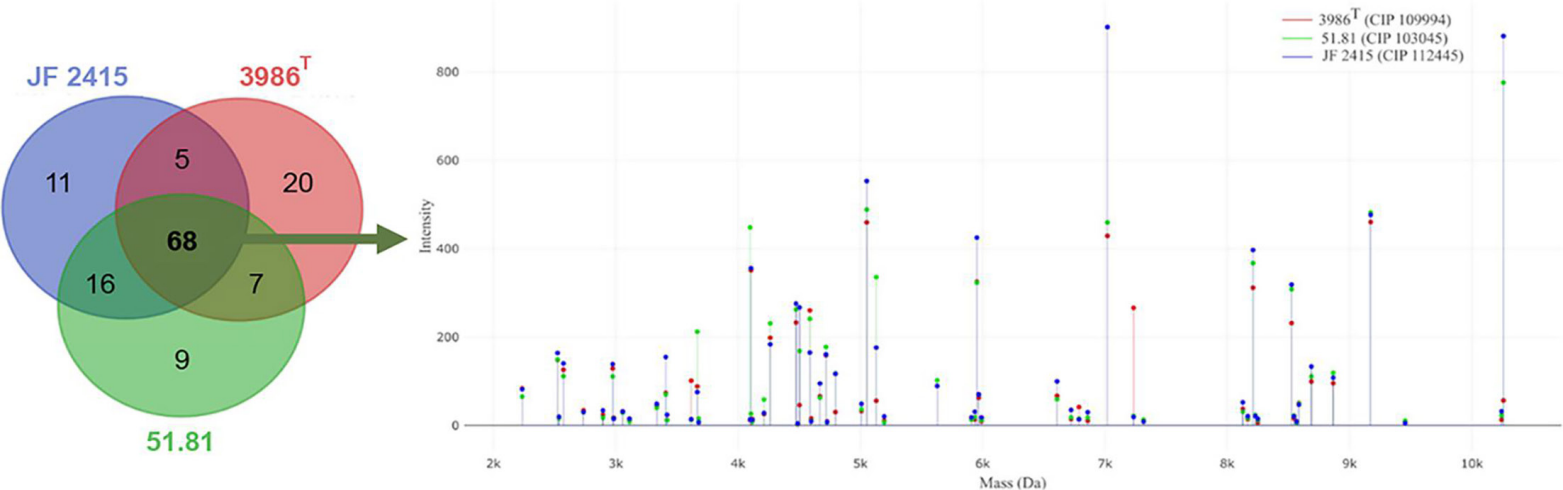
Venn diagram representing the shared and unique peaks identified in the MALDI-TOF MS spectra of strains 3896^T^, 51.81 and JF2415, and the spectra represent the core peaks (peaks shared by all three strains).

The MALDI-TOF MS spectra of strains 3986^T^, 51.81, and JF 2415 did not yield a reliable identity when searched against the biotyper database (version 12.0). A total of 136 peaks were identified after aligning the spectra of the three strains, with 68 peaks being shared among all strains (Table S8 and Fig. 6). Additionally, 20 peaks were unique to strain 3986^T^, nine to strain 51.81, and 11 to strain JF 2415. Notably, strains 51.81 and JF 2415 exhibited a higher number of shared peaks between them than with strain 3986^T^, which aligns with the observed genetic similarities. The shared peaks within the strains of this new species hold promise for developing a MALDI-TOF MS biotyper-based diagnosis. Several studies have suggested that improving the spectral database can enhance the identification rate of MALDI-TOF MS-based identification, establishing it as the primary tool for microbial identification, applicable not only to clinical microbes but also beyond [38,39].

### Phenotypic features

Strains 3986^T^, 51.81 and JF 2415 grew well on TSA, Columbia agar with 10 % horse blood and BHI. Colonies on TSA appeared small, round, shiny and translucent (Figs S3 and S4). All strains formed aggregates in BHI broth, exhibiting different patterns such as pellicle formation on the top of the broth for strain 3986^T^, sediment formation at the bottom for strain 51.81, or both for strain JF 2415. The cells appeared coccobacilli or diplococcobacilli under bright-field microscope and in transmission electron microscope images (Figs 5, S2 and S4). A recent study proposed that the rod shape was the ancestral form of the family *Neisseriaceae*. Cocci emerged independently within the genus *Neisseria* on two occasions, while multicellular morphologies were observed in bacteria belonging to the genera *Alysiella*, *Simonsiella* and *Conchiformibius* [6]. Cell size varied from 1 to 2 µm. The optimal temperature for growth on TSA was 37 °C (range, 28–45 °C) and the optimal pH was 8.5 (range, pH 5.5–10 with only slight growth at pH 5.5). Growth was equally fast with or without 5 % CO_2_ and strains 3986^T^ and 51.81 grew with 0.5 % oxgall added to TSB (not tested for strain JF 2415), which coincided with the discovery of one of the strains in the liver of a rabbit, where bile salts are highly concentrated. Growth occurred in microaerophilic conditions but not in anaerobic conditions. Strains 3986^T^, 51.81 and JF 2415 were Gram-negative, non-motile and non-hemolytic. They were catalase and oxidase positive.

Strains 3986^T^, 51.81 and JF 2415 showed indole production while the other strains DSM 23338^T^ (*N. bacilliformis*), CIP 108935^T^ (*K. potus*), CIP 106968^T^ (*N. dentiae*), CIP 72.27^T^ (*N. elongata*), and CIP 79.18^T^ (*N. gonorrhoeae*) did not produce indole (Table 1). Indole serves as intracellular, interspecies and even interkingdom signalling molecule [62]. Indole production appears a very distinctive feature for strains 3986^T^, 51.81 and JF 2415 as a blastn search of UniProt protein P0A853 (tryptophanase of *Escherichia coli* strain K12) against the reference dataset resulted only in the genomes of the three strains above 50 % identity (with respectively 81.1 and 81.3 % identities). Strains 3986^T^ and 51.81 were positive for lipase C14 in the API ZYM test (Table S9), but strain JF 2415 was found to be lipase C14 negative. The search for antimicrobial resistance genes by card in the genome sequences of the three strains returned only one strict hit, the antibiotic efflux pump gene qacJ found in JF 2415. Nevertheless, antibiograms (Table S10) proved that both 3986^T^ and 51.81 were contact resistant to lincomycin and vancomycin, which are established intrinsic resistances of *Neisseria* species according to CA-SFM/EUCAST [63]. On the contrary, the intrinsic resistance to trimethoprim was not found in any strain, while strain 51.81 was contact resistant to streptomycin.

Based on the literature and the various analyses performed in this study, including 16S rRNA and core gene phylogenies, and the distribution of ANI, AAI and DUS values, it is suggested that taxonomic reclassification and/or reunification may be needed for several taxa (genera and species) within the family *Neisseriaceae*. Specifically, the reunification of *Bergeriella denitrificans* and the reclassification of *Kingella potus*, *Morococcus cerebrosus*, *Uruburuella testudinis*, and *U. suis* within the genus *Neisseria*, along with the reclassification of *N. shayeganii* within the genus *Eikenella*, require immediate attention. However, such taxonomic changes, especially for taxa with clinical relevance, should undergo detailed discussions and require consensus within the scientific community. It is proposed that a consensus-seeking discussion involving concerned parties related to the family *Neisseriaceae*, particularly the genera *Eikenella* and *Neisseria*, should deliberate on the circumscription of these genera within the family *Neisseriaceae*. The ICSP subcommittee for the family *Neisseriaceae* should be reconstituted and we would be interested in its reconstitution with other interested parties to offer a consensus-based solution.

Meanwhile, based on genetic and phenotypic characteristics, strains 3986^T^, 51.81 and JF 2415 represent a novel species within the genus *Neisseria*, for which we propose the name *Neisseria leonii* sp. nov., with 3986^T^ designated as the type strain.

## Description of *Neisseria leonii* sp. nov.

*Neisseria leonii* (le.o’ni.i. N.L. gen. n. *leonii,* of Léon, in honour of Léon Boutroux (1851–1921), a French chemist who worked under the direction of Louis Pasteur on glucose fermentation. He notably published a book on the chemistry of bread making in 1897).

Cells are diplococcobacilli with length around 1–2 µm. Gram-negative, non-motile and non-haemolytic. Colonies are small, round, shiny and translucent. Cultures grow well on TSA, Columbia agar with 10 % horse blood or BHI agar. Optimal growth temperature is at 37 °C and pH 8.5 and can tolerate bile salts at least up to 0.5 %. Growth with or without 5 % CO_2_ and in microaerophilic conditions. No growth in anaerobic conditions. Oxidase and catalase positive. Positive for production of indole, leucine arylamidase, acid phosphatase and naphthol-AS-BI-phosphohydrolase. Negative for production of trypsin, α-chymotrypsin, α-galactosidase, β-galactosidase, β-glucuronidase, α-glucosidase, β-glucosidase, *N*-acetyl-β-glucosaminidase, α-mannosidase and α-fucosidase. Negative for utilization of d-glucose, d-fructose, maltose and sucrose. The DNA G+C content of the type strain is 56.92 mol%.

The type strain of the species is 3986^T^ (=R726^T^=CIP 109994^T^=LMG 32907^T^). It was isolated from the liver of a rabbit in 1972 in the Institut Pasteur of Lyon, France. The GenBank accession number of the whole genome sequence is CP145606.1 and the 16S rRNA gene sequence accession number is OQ121838.1.

## supplementary material

10.1099/ijsem.0.006460 Uncited Fig. S1.

10.1099/ijsem.0.006460 Uncited Table S1.

## Abbreviations

AAI: average amino acid identity
ANI: average nucleotide identity
ASV: amplicon sequence variant
BHI: brain heart infusion
CDS: coding sequence
dDDH: digital DNA–DNA hybridization
DUS: DNA uptake sequence
GTDB: Genome Taxonomy Database
ICSP: International Committee on Systematics of Prokaryotes
LPSN: List of Prokaryotic Names with Standing in Nomenclature
MALDI-TOF MS: matrix-assisted laser desorption/ionization time-of-flight mass spectrometry
ML: maximum-likelihood
MP: maximum-parsimony
MSP: mean spectral profile
NJ: neighbour-joining
PGAP: Prokaryotic Genome Annotation Pipeline
RM: restriction-modification
rMLST: ribosomal multilocus sequence typing
TSA: trypticase soy agar
TSB: tryptone soy broth
TYGS: Type Strain Genome Server
