# Supplementary material for: Neisseria leonii sp. nov., isolated from the nose, lung, and liver of rabbits
Source: Int J Syst Evol Microbiol. 2024 Jul 18;74(7):006460. doi: 10.1099/ijsem.0.006460 (PMC11316581; doi:10.1099/ijsem.0.006460)
Supplement: Uncited Fig. S1. [file ijsem-74-06460-s001.pdf]

Supplementary material

Title: *Neisseria leonii* sp. *nov*. isolated from the nose, lung, and liver of rabbits.

Authors and affiliations:

Martin Boutroux[1-2*], Sandrine Favre-Rochex[3], Olivier Gorgette[4], Gérald Touak[3], Estelle Mühle[3], Christiane Bouchier[3], Olivier Chesneau[3], Frédéric J. Veyrier[5], Dominique Clermont[3], Praveen Rahi[3*]

[1]Institut Pasteur, Université Paris Cité, Center of Biological Resources of Institut Pasteur (CRBIP), 75015 Paris, France

[2]Current address: River Ecosystems Laboratory, École Polytechnique Fédérale de Lausanne, CH-1015 Lausanne, Switzerland

[3]Institut Pasteur, Université Paris Cité, Collection of Institut Pasteur (CIP), 75015 Paris, France

[4]Institut Pasteur, Université Paris Cité, Ultrastructural BioImaging Unit, 75015 Paris, France

[5]INRS-Centre Armand-Frappier Santé Biotechnologie, Bacterial Symbionts Evolution, Laval, Quebec H7V 1B7, Canada

Corresponding authors:

Martin Boutroux, martin.boutroux@epfl.ch, martinboutroux@outlook.fr
Praveen Rahi, praveen.rahi@pasteur.fr, praveen_rahi22@yahoo.co.in

Supplementary text 1: Metagenomic analysis between strain 3986^T with public microbiome rabbit datasets

An ASV present 19 times in one of the 126 samples (SRR6475038) is 100% identical to the sequence of strain 3986^T. The characteristics of this ASV were:

Run: SRR6475038 from SRX3564915

BioSample: SAMN08358139

**host**	Oryctolagus cuniculus
**collection date**	2015-11-06
**isolation source**	feces
**geographic location**	Spain:Rabanales, Cordoba
**latitude and longitude**	missing
**sex**	female
**age**	> 2 years
**host_subsp**	cuniculus
**individual**	P.44
**origin**	P
**plot**	6
**warren**	8
**cage**	NA
**weight**	1374
**tarsus**	61.74
**fate**	alive

Sequence:

TACGTAGGGTGCGAGCGTTAATCGGAATTACTGGGCGTAAAGCGGGCGCAGACGGTTTGTTAAG
CAGGATGTGAAATCCCCGGGCTCAACCTGGGAACTGCGTTCTGAACTGGCAGGCTAGAGTGTGT
CAGAGGGGGGTAGAATTCCACGTGTAGCAGTGAAATGCGTAGAGATGTGGAGGAATACCGATGG
CGAAGGCAGCCCCCTGGGATAACACTGACGTTCATGCCCGAAAGCGTGGGTAGCAAACAGG

A similar search for identical sequences in the fecal microbiome of different herbivores among which rabbits (SRP405849) (no publication associated yet) resulted in zero BLAST hit, confirming the rarity of the new *Neisseria* species.

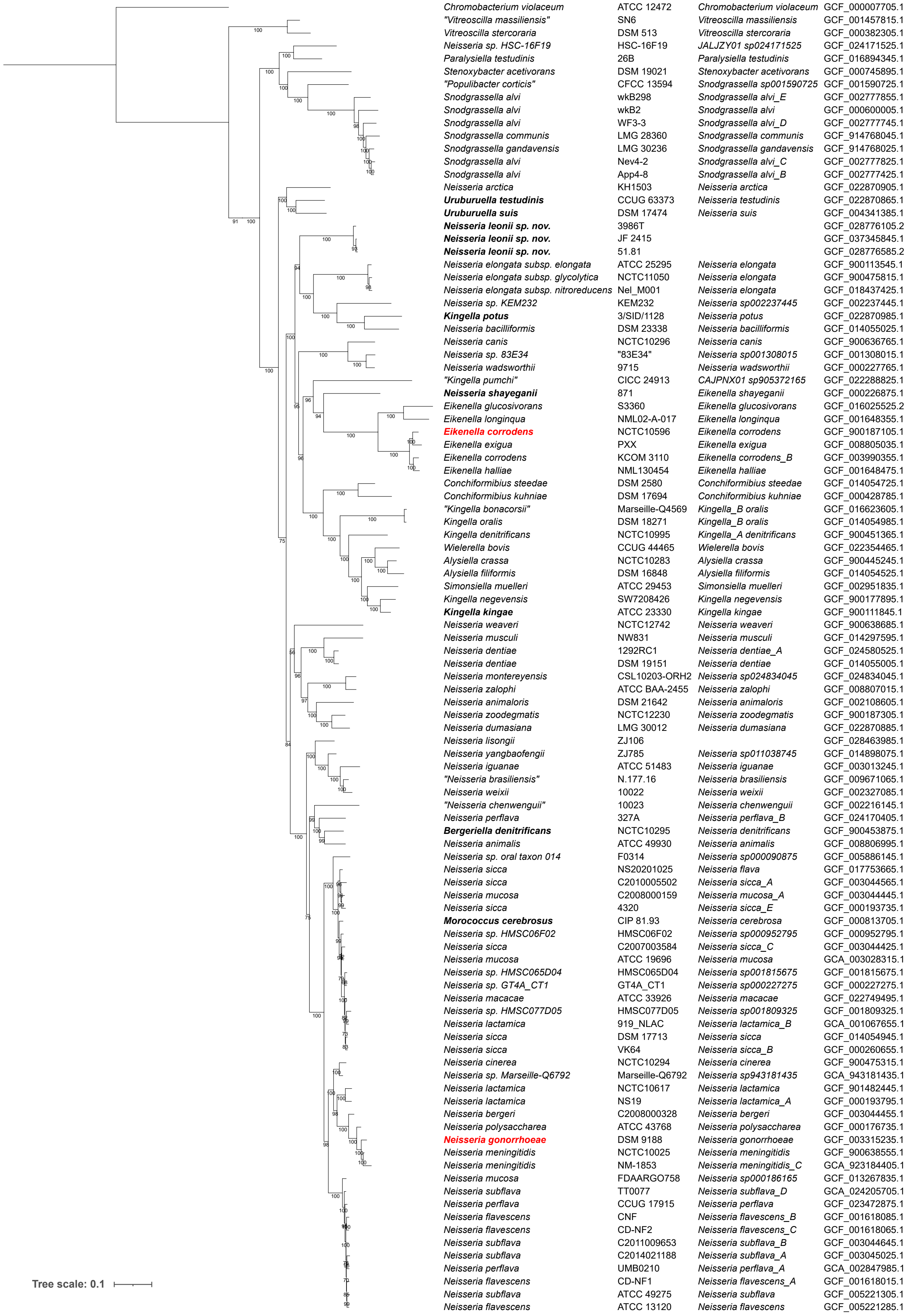
Chromobacterium violaceum ATCC 12472 Chromobacterium violaceum GCF_000007705.1
"Vitreoscilla massiliensis" SN6 Vitreoscilla massiliensis GCF_001457815.1
Vitreoscilla stercoraria DSM 513 Vitreoscilla stercoraria GCF_000382305.1
Neisseria sp. HSC-16F19 HSC-16F19 JALJZY01 sp024171525 GCF_024171525.1
Paralysiella testudinis 26B Paralysiella testudinis GCF_016894345.1
Stenoxybacter acetivorans DSM 19021 Stenoxybacter acetivorans GCF_000745895.1
"Populibacter corticis" CFCC 13594 Snodgrassella sp001590725 GCF_001590725.1
Snodgrassella alvi wkB298 Snodgrassella alvi_E GCF_002777855.1
Snodgrassella alvi wkB2 Snodgrassella alvi GCF_000600005.1
Snodgrassella alvi WF3-3 Snodgrassella alvi_D GCF_002777745.1
Snodgrassella communis LMG 28360 Snodgrassella communis GCF_914768045.1
Snodgrassella gandavensis LMG 30236 Snodgrassella gandavensis GCF_914768025.1
Snodgrassella alvi Nev4-2 Snodgrassella alvi_C GCF_002777825.1
Snodgrassella alvi App4-8 Snodgrassella alvi_B GCF_002777425.1
Neisseria arctica KH1503 Neisseria arctica GCF_022870905.1
Uruburuella testudinis CCUG 63373 Neisseria testudinis GCF_022870865.1
Uruburuella suis DSM 17474 Neisseria suis GCF_004341385.1
Neisseria leonii sp. nov. 3986T GCF_028776105.2
Neisseria leonii sp. nov. JF 2415 GCF_037345845.1
Neisseria leonii sp. nov. 51.81 GCF_028776585.2
Neisseria elongata subsp. elongata ATCC 25295 Neisseria elongata GCF_900113545.1
Neisseria elongata subsp. glycolytica NCTC11050 Neisseria elongata GCF_900475815.1
Neisseria elongata subsp. nitroreducens Nel_M001 Neisseria elongata GCF_018437425.1
Neisseria sp. KEM232 KEM232 Neisseria sp002237445 GCF_002237445.1
Kingella potus 3/SID/1128 Neisseria potus GCF_022870985.1
Neisseria bacilliformis DSM 23338 Neisseria bacilliformis GCF_014055025.1
Neisseria canis NCTC10296 Neisseria canis GCF_900636765.1
Neisseria sp. 83E34 "83E34" Neisseria sp001308015 GCF_001308015.1
Neisseria wadsworthii 9715 Neisseria wadsworthii GCF_000227765.1
"Kingella pumchi" CICC 24913 CAJPNX01 sp905372165 GCF_022288825.1
Neisseria shayeganii 871 Eikenella shayeganii GCF_000226875.1
Eikenella glucosivorans S3360 Eikenella glucosivorans GCF_016025525.2
Eikenella longinqua NML02-A-017 Eikenella longinqua GCF_001648355.1
Eikenella corrodens NCTC10596 Eikenella corrodens GCF_900187105.1
Eikenella exigua PXX Eikenella exigua GCF_008805035.1
Eikenella corrodens KCOM 3110 Eikenella corrodens_B GCF_003990355.1
Eikenella halliae NML130454 Eikenella halliae GCF_001648475.1
Conchiformibius steedae DSM 2580 Conchiformibius steedae GCF_014054725.1
Conchiformibius kuhniae DSM 17694 Conchiformibius kuhniae GCF_000428785.1
"Kingella bonacorsii" Marseille-Q4569 Kingella_B oralis GCF_016623605.1
Kingella oralis DSM 18271 Kingella_B oralis GCF_014054985.1
Kingella denitrificans NCTC10995 Kingella_A denitrificans GCF_900451365.1
Wielerella bovis CCUG 44465 Wielerella bovis GCF_022354465.1
Alysiella crassa NCTC10283 Alysiella crassa GCF_900445245.1
Alysiella filiformis DSM 16848 Alysiella filiformis GCF_014054525.1
Simonsiella muelleri ATCC 29453 Simonsiella muelleri GCF_002951835.1
Kingella negevensis SW7208426 Kingella negevensis GCF_900177895.1
Kingella kingae ATCC 23330 Kingella kingae GCF_900111845.1
Neisseria weaveri NCTC12742 Neisseria weaveri GCF_900638685.1
Neisseria musculi NW831 Neisseria musculi GCF_014297595.1
Neisseria dentiae 1292RC1 Neisseria dentiae_A GCF_024580525.1
Neisseria dentiae DSM 19151 Neisseria dentiae GCF_014055005.1
Neisseria montereyensis CSL10203-ORH2 Neisseria sp024834045 GCF_024834045.1
Neisseria zalophi ATCC BAA-2455 Neisseria zalophi GCF_008807015.1
Neisseria animaloris DSM 21642 Neisseria animaloris GCF_002108605.1
Neisseria zoodegmatis NCTC12230 Neisseria zoodegmatis GCF_900187305.1
Neisseria dumasiana LMG 30012 Neisseria dumasiana GCF_022870885.1
Neisseria lisongii ZJ106 GCF_028463985.1
Neisseria yangbaofengii ZJ785 Neisseria sp011038745 GCF_014898075.1
Neisseria iguanae ATCC 51483 Neisseria iguanae GCF_003013245.1
"Neisseria brasiliensis" N.177.16 Neisseria brasiliensis GCF_009671065.1
Neisseria weixii 10022 Neisseria weixii GCF_002327085.1
"Neisseria chenwenguii" 10023 Neisseria chenwenguii GCF_002216145.1
Neisseria perflava 327A Neisseria perflava_B GCF_024170405.1
Bergeriella denitrificans NCTC10295 Neisseria denitrificans GCF_900453875.1
Neisseria animalis ATCC 49930 Neisseria animalis GCF_008806995.1
Neisseria sp. oral taxon 014 F0314 Neisseria sp000090875 GCF_005886145.1
Neisseria sicca NS20201025 Neisseria flava GCF_017753665.1
Neisseria sicca C2010005502 Neisseria sicca_A GCF_003044565.1
Neisseria mucosa C2008000159 Neisseria mucosa_A GCF_003044445.1
Neisseria sicca 4320 Neisseria sicca_E GCF_000193735.1
Morococcus cerebrosus CIP 81.93 Neisseria cerebrosa GCF_000813705.1
Neisseria sp. HMSC06F02 HMSC06F02 Neisseria sp000952795 GCF_000952795.1
Neisseria sicca C2007003584 Neisseria sicca_C GCF_003044425.1
Neisseria mucosa ATCC 19696 Neisseria mucosa GCA_003028315.1
Neisseria sp. HMSC065D04 HMSC065D04 Neisseria sp001815675 GCF_001815675.1
Neisseria sp. GT4A_CT1 GT4A_CT1 Neisseria sp000227275 GCF_000227275.1
Neisseria macacae ATCC 33926 Neisseria macacae GCF_022749495.1
Neisseria sp. HMSC077D05 HMSC077D05 Neisseria sp001809325 GCF_001809325.1
Neisseria lactamica 919_NLAC Neisseria lactamica_B GCA_001067655.1
Neisseria sicca DSM 17713 Neisseria sicca GCF_014054945.1
Neisseria sicca VK64 Neisseria sicca_B GCF_000260655.1
Neisseria cinerea NCTC10294 Neisseria cinerea GCF_900475315.1
Neisseria sp. Marseille-Q6792 Marseille-Q6792 Neisseria sp943181435 GCA_943181435.1
Neisseria lactamica NCTC10617 Neisseria lactamica GCF_901482445.1
Neisseria lactamica NS19 Neisseria lactamica_A GCF_000193795.1
Neisseria bergeri C2008000328 Neisseria bergeri GCF_003044455.1
Neisseria polysaccharea ATCC 43768 Neisseria polysaccharea GCF_000176735.1
Neisseria gonorrhoeae DSM 9188 Neisseria gonorrhoeae GCF_003315235.1
Neisseria meningitidis NCTC10025 Neisseria meningitidis GCF_900638555.1
Neisseria meningitidis NM-1853 Neisseria meningitidis_C GCA_923184405.1
Neisseria mucosa FDAARGO758 Neisseria sp000186165 GCF_013267835.1
Neisseria subflava TT0077 Neisseria subflava_D GCA_024205705.1
Neisseria perflava CCUG 17915 Neisseria perflava GCF_023472875.1
Neisseria flavescens CNF Neisseria flavescens_B GCF_001618085.1
Neisseria flavescens CD-NF2 Neisseria flavescens_C GCF_001618065.1
Neisseria subflava C2011009653 Neisseria subflava_B GCF_003044645.1
Neisseria subflava C2014021188 Neisseria subflava_A GCF_003045025.1
Neisseria perflava UMB0210 Neisseria perflava_A GCA_002847985.1
Neisseria flavescens CD-NF1 Neisseria flavescens_A GCF_001618015.1
Neisseria subflava ATCC 49275 Neisseria subflava GCF_005221305.1
Neisseria flavescens ATCC 13120 Neisseria flavescens GCF_005221285.1
Tree scale: 0.1

Figure S1: Phylogenetic tree obtained in IQ-TREE from the alignment of rMLST alleles of strains 3986^T, 51.81, JF 2415 and 100 type strains of the *Neisseriaceae* family with maximum likelihood method. Each line contains the isolate name, the accession number, the name of the species according to LPSN and according to GTDB if it differs. The taxonomy of these strains and of strains 51.81 and JF 2415 is in bold to highlight them. Type species of *Neisseria* and *Eikenella* are colored in red as well. GTR+F+I+R6 model was chosen according to BIC by ModelFinder. *Chromobacterium violaceum* ATCC 12472^T was used as outgroup. Bootstrap values above 50% are displayed. Bar, 0.1 changes per site.

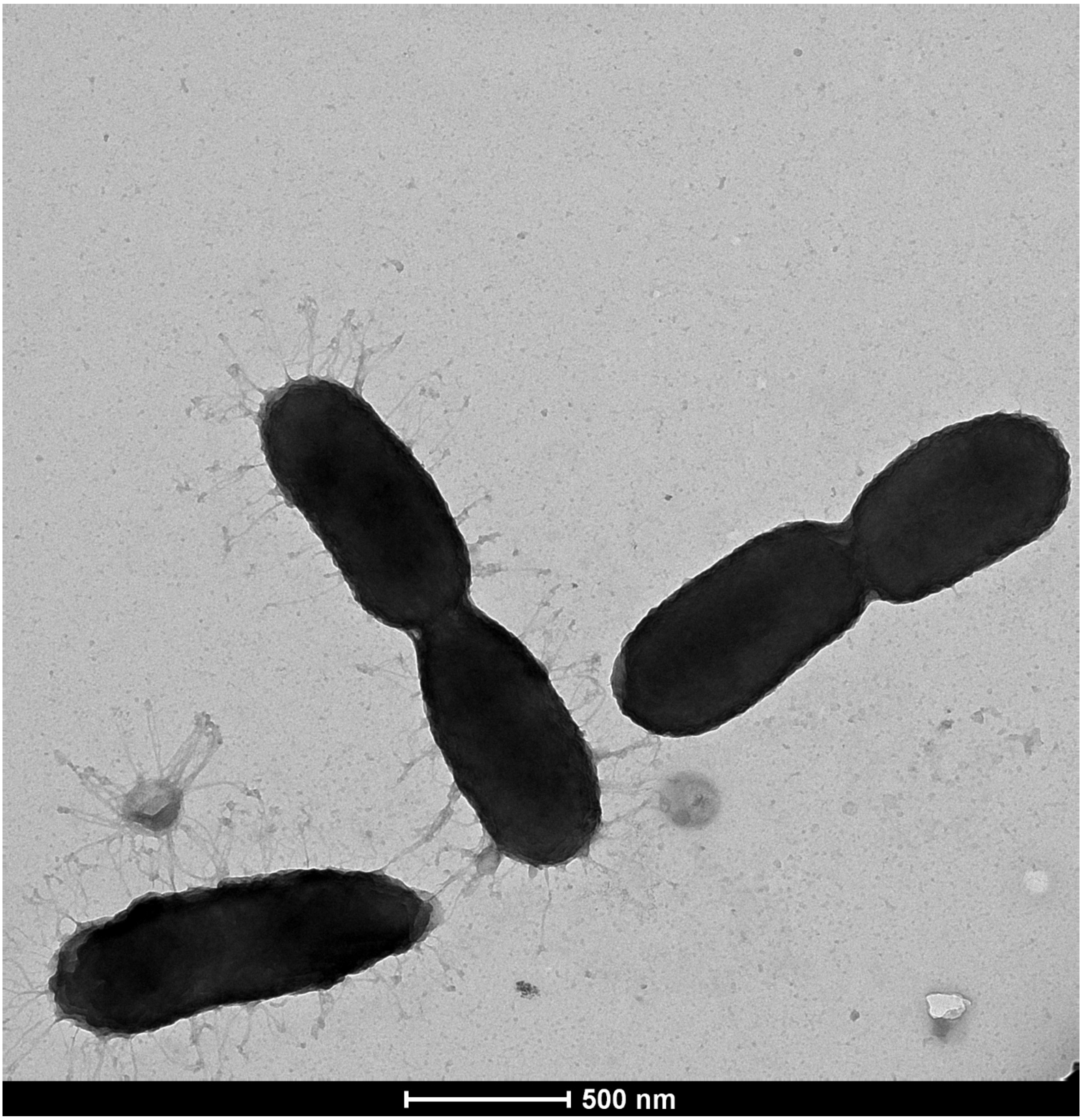

Figure S2: Transmission electron microscopy image of strain 3986^T. Two pairs of diplococcobacilli and one coccobacillus appears connected by Tfp-like fibers. Scale bar 500 nm.

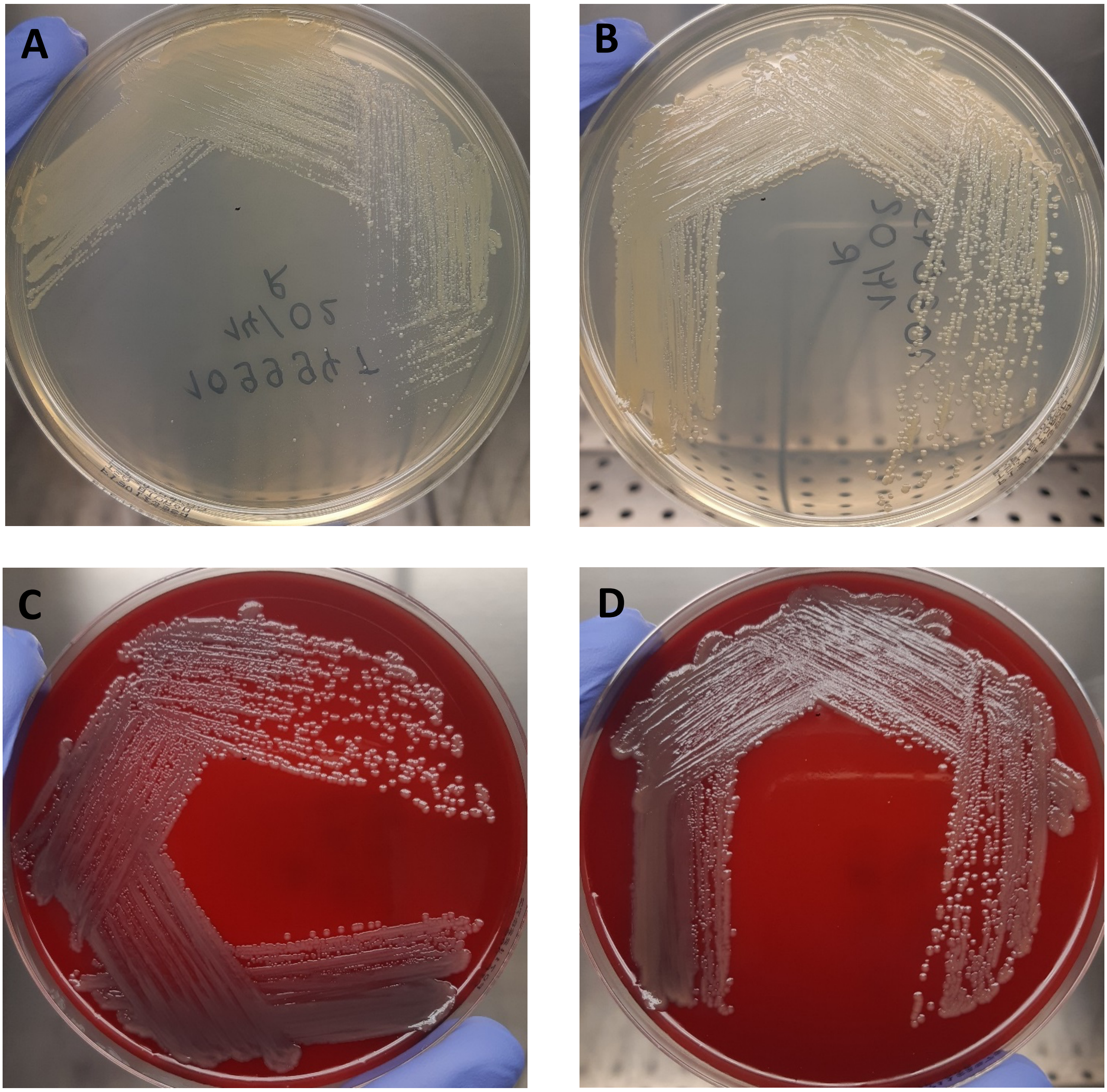

Figure S3: Colonies of strains 3896^{T} and 51.81 after 24 hours incubation at 37°C as observed on (a, b) Trypticase Soy Agar; (c, d) Columbia agar with 10 % horse blood.

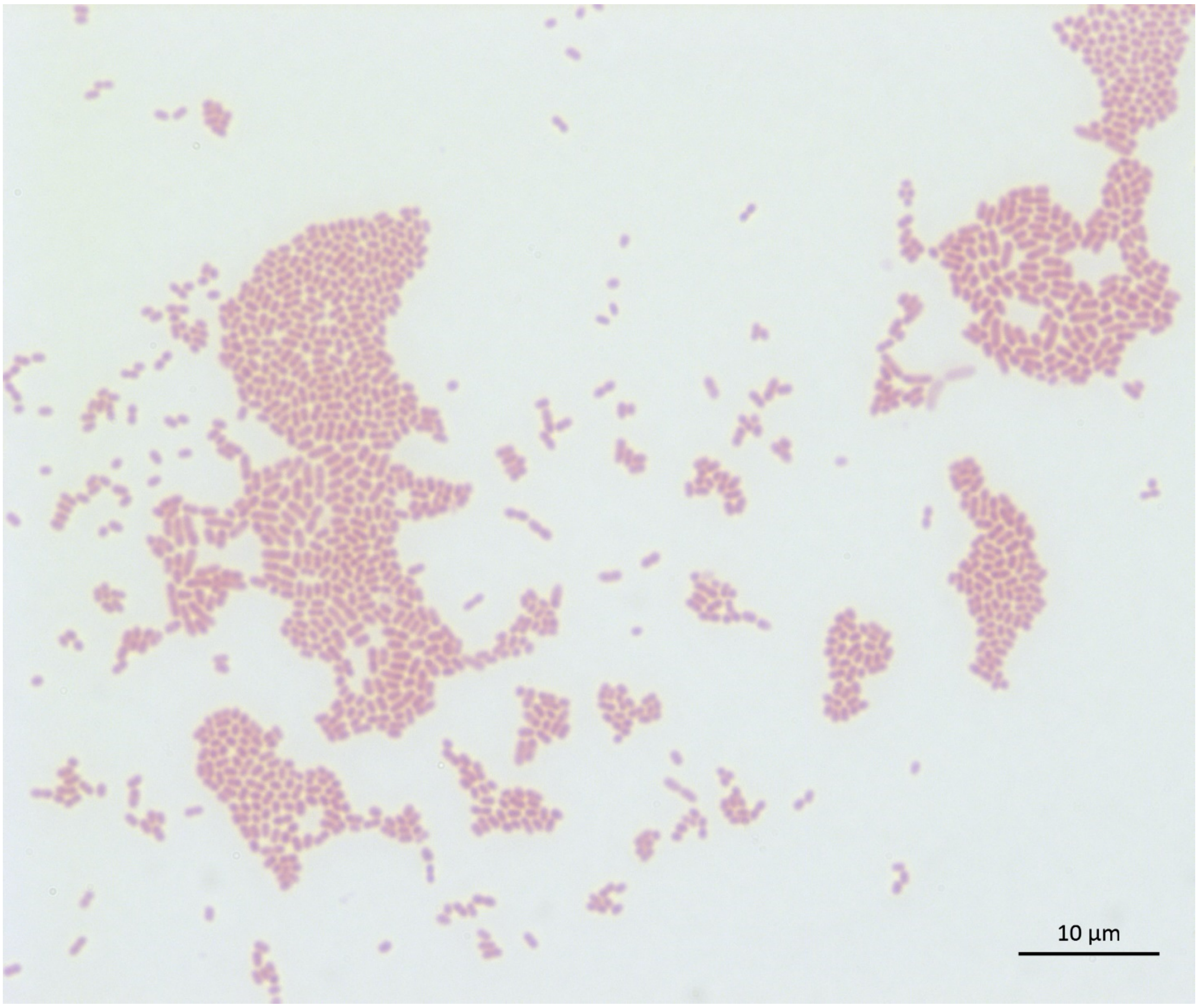

Figure S4: Image obtained under an optical microscope at magnification 100X of the strain 3986^T. Cells are Gram-negative and form clear aggregates. Scale bar 10 µm.
